# Laboratory Tests in Inflammatory Bowel Disease: An Evidence-Based Approach to Daily Practice

**DOI:** 10.3390/biomedicines13020491

**Published:** 2025-02-17

**Authors:** Katelin Durham, Tyler Atagozli, David E. Elliott, M. Nedim Ince

**Affiliations:** 1 Division of Gastroenterology and Hepatology, Department of Internal Medicine, University of Iowa, 200 Hawkins Drive, Iowa City, IA 52242, USA; katelin-durham@uiowa.edu (K.D.); david-elliott@uiowa.edu (D.E.E.); 2 Iowa City Veterans Affairs Medical Center, 601 Highway 6 W, Iowa City, IA 52246, USA; 3 Carver College of Medicine, University of Iowa, 375 Newton Road, Iowa City, IA 52242, USA; tyler-atagozli@uiowa.edu

**Keywords:** inflammatory bowel disease, ulcerative colitis, Crohn’s disease, therapeutic drug monitoring, anti-drug antibodies, trough level

## Abstract

Inflammatory bowel diseases (IBDs) comprise a group of chronic gastrointestinal disorders characterized by periods of relapse and remission. The mainstay of treatment is medical, involving medications such as steroids, immune modulators, monoclonal antibodies (categorized as biologics), and small molecules. These medications can provide profound therapeutic benefits, but they can also cause severe and irreversible toxicities. Clinicians may utilize laboratory tests in the diagnosis and management of IBD including assessment of disease activity, monitoring medication response or toxicity, surveillance of infectious complications, and detection of nutritional deficiencies. Routine use of laboratory tests may help clinicians avoid reactivation of life-threatening infections such as tuberculosis or hepatitis B virus upon initiation of immune suppressive therapy. They can also be used to detect vitamin deficiencies such as B12 deficiency, which has the potential to cause irreversible neurologic damage. While some laboratory tests constitute established practices, the utility of newer tests such therapeutic drug monitoring (TDM) in the era of biologics is an evolving topic. Although clinical assessment with imaging, endoscopic, and histopathological examination is standard practice, laboratory tests serve as valuable adjuncts. We aim to explore the broad range of laboratory tests available to clinicians and to summarize their application in the current management of IBD in daily clinical practice, with special attention to updates in therapeutic drug monitoring.

## 1. Introduction

Inflammatory bowel diseases (IBDs) comprise a group of chronic gastrointestinal disorders characterized by periods of relapse and remission. Although IBD consists mainly of two diseases, ulcerative colitis (UC) and Crohn’s disease (CD), 5–15% of cases cannot be grouped as either and are designated as indeterminate colitis [1]. Certain other forms of colitides—for example, microscopic colitis, which consists of lymphocytic and collagenous varieties—can also be grouped within the framework of IBD. UC and CD usually affect young adults with a second peak of increased frequency after the age of 50 years, although IBD can also be a childhood disease or present as a severe disorder in infants [2,3]. The etiopathogenesis of IBD is complex but may be summarized as the stimulation of gut inflammation by environmental factors in the right genetic background [4].

**Figure 1:**
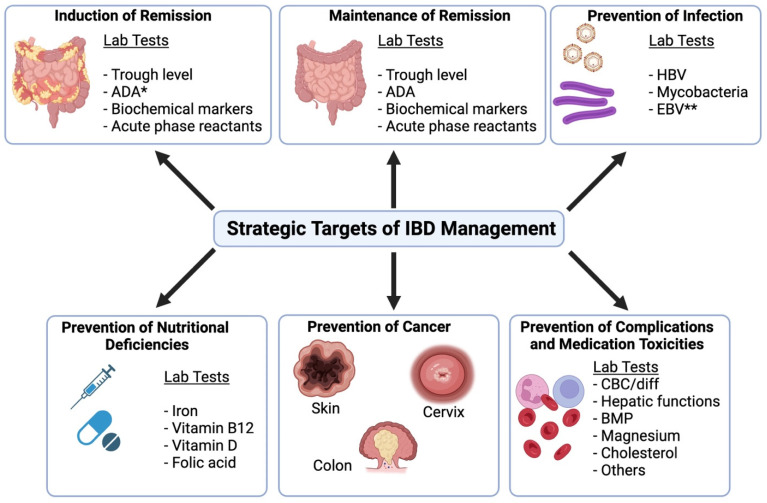
Strategic targets of IBD therapy and useful laboratory tests associated with these targets. * ADA: Anti-drug antibody; ** screening for EBV infection is only recommended in pediatric patients before starting thiopurine therapy.

The management of IBD involves the development of short- and long-term treatment strategies, which can be summarized as strategic targets (Figure 1) and are as follows:

1. *Induction of remission.* In a patient who presents with an acute flare, it is essential to induce clinical and endoscopic remission and assess patients for life-threatening complications such as toxic megacolon or unresponsiveness to medications. Steroids constitute the mainstay of treatment, although further treatment with 5-aminosalycilic acid (5-ASA) compounds, immune modulators, biologics, or small-molecule drugs may also be indicated.
2. *Maintenance of remission.* Patients without symptoms or signs of active IBD are kept on maintenance medications, such as immune modulators or biologics, to prevent relapse and achieve better symptom control. Laboratory tests may be used to confirm biochemical remission and for the surveillance of disease activity.
3. *Prevention of infections.* Treatment with immune suppressive medications can be associated with severe and life-threatening infectious complications. These include opportunistic infections like pneumocystis pneumonia and the reactivation of chronic latent infections like tuberculosis or hepatitis B virus (HBV). Laboratory tests are useful in the prevention, diagnosis, and management of these infectious complications.
4. *Prevention of nutritional deficiencies.* Severe intestinal inflammation can lead to chronic blood loss and malabsorption, resulting in deficiencies of essential vitamins and minerals. Detection and replacement of nutritional deficiencies is essential to prevent complications and improve overall well-being.
5. *Prevention of cancer.* IBD predisposes patients to cancer, such as colon cancer in patients with long-standing (>10 years) diagnosis and with >30% involvement of the colon. Chronic immune suppression, as part of IBD treatment, also predisposes patients to skin cancer, carcinoma of the cervix, and lymphoma. Laboratory tests provide limited benefit to the prevention of cancer in IBD, although routine Pap smears in sexually active females are included in the standard-of-care. Pap smears are not the topic of our current review article.
6. *Prevention of complications and medication toxicity.* IBD and the therapeutic agents used for its treatment predispose patients to extraintestinal organ damage or even organ failure. Some conditions, like the development of arthritis or skin disease, may be monitored clinically, whereas diagnosing renal and hepatobiliary diseases may require routine laboratory monitoring.

While endoscopy and imaging are quintessential in the management of IBD, laboratory tests may be useful adjuncts. An expanding array of laboratory tests are now available to aid in diagnosis, assessment of disease activity, monitoring for medication response or toxicity, surveillance of infectious complications, and detection of nutritional deficiencies. In different settings, laboratory tests provide crucial reference in the assessment of the patient and the response to therapy. Herein, our objectives are to summarize the application of laboratory tests in the current management of IBD, including utility of genetic testing in clinical practice or use of seromarkers, with special attention to therapeutic drug monitoring. We also discuss how we use laboratory tests to diagnose infections, screening for vitamin or iron deficiency in IBD, where inflammation is triggered by environmental factors in the setting of genetic predisposition.

## 2. Genetic, Serologic, and Multiomic Testing in IBD

Genome-wide association studies and next-generation sequencing studies have identified more than 240 susceptibility loci associated with IBD [5]. One of the strongest associations is the presence of CARD15/NOD2 mutations in CD. CARD15/NOD2 is the cellular receptor of bacterial muramyl dipeptide. The expression of CARD15/NOD2 variants results in abnormal inflammatory gene expression in response to stimulation by the inflammatory mediators of the gut microbiome and also impaired autophagy [6]. CARD15/NOD2 mutations impair NF-kB signaling pathways and cytokine responses to pathogens. Mono-allelic CARD15/NOD2 mutation carriers have a two- to four-fold increased risk of developing CD, while bi-allelic mutation carriers are associated with 20- to 40-fold increased risk for CD [7,8]. However, CARD15/NOD2 mutations occur in up to 2% of the general population, and approximately 60% of CD patients will have no mutation detected [9]. Regarding prognostic significance of the CARD15/NOD2 mutations, in one meta-analysis the presence of bi-allelic CARD15/NOD2 mutations among CD patients was found to have 98% specificity for a complicated disease course involving fistulas and strictures [10]. As CARD15/NOD2 mutations may also cause steroid refractoriness or increase the risk for surgical therapy, experts have suggested that more intensive therapy should be considered for these high-risk patients with CARD15/NOD2 mutations, although a decision for such a practice in clinical routine will require further evidence from randomized controlled trials [6,7]. Furthermore, certain HLA allotypes have been associated with IBD, with the most notable being HLA-DRB1*0103, which is linked to the development of both UC and CD, as well as extraintestinal manifestations like arthropathy, erythema nodosum, and uveitis [11]. In addition, the HLA-DQA1*05 allele is associated with two-fold risk of antibody formation and secondary loss of response to anti-TNF drugs. In such patients, routine drug monitoring or an alternative therapy to anti-TNF class drugs may be considered [12]. Additional mutations in genes associated with autophagy (ATG16L1, IRGM, LRRK2), pathogen recognition (CARD9), lymphocyte differentiation (IL23R), anti-inflammatory interleukins (IL-10), and pro-inflammatory cytokines (TNFSF15) have also been implicated in the development of IBD [13]. Clearly, the pathophysiology of IBD is complex with genetics only contributing partially, along with epigenetic and environmental factors.

Genetic testing is generally not recommended for adult-onset IBD but should be considered in cases of infantile IBD, in which diagnosis is made before 2 years of age, and very early onset IBD, in which diagnosis is made before 6 years of age [14]. In these patients, monogenetic etiologies are more common and often involve a primary immune deficiency, which may be cured with other therapeutic approaches, such as bone marrow transplantation. Nearly 60 genetic mutations have been identified to be associated with monogenic IBD, including IL-10, IPEX, WAS, and XIAP [3]. Suspected cases may be diagnosed through a combination of immune and genetic testing.

Serological markers associated with CD and UC have been reported to have limited utility in clinical practice. The most studied antibodies are antineutrophil cytoplasmic antibody with an atypical perinuclear pattern (pANCA), which has 55.3% sensitivity (95% CI: 53–58%) and 88.5% specificity (95% CI: 87–90%), for UC and anti-*Saccharomyces cerevisiae* antibody (ASCA), which has 54.6% sensitivity (95% CI: 52–59%) and 92.8% specificity (95% CI: 90–95%), for CD [15]. Given overall low sensitivity, serology cannot exclude the presence of IBD but may be helpful for predicting clinical course in patients with indeterminate colitis. In UC patients, the presence of pANCA has been associated with a higher risk for developing chronic pouchitis after colectomy and the creation of an ileoanal pouch (OR: 1.76, 95% CI: 1.19–2.61, *p* < 0.01) [16]. Thus, the presence of positive pANCA serology may impact the surgical decision to create an end-ileostomy over an ileoanal pouch. In contrast, the presence of ASCA has been associated with a higher frequency of ileal involvement, a more severe disease course, and a greater need for immunosuppression in CD [17]. Despite their relative specificity among patients with IBD, these serologies have poor specificity in the general population and should not be used to screen for IBD. Routine use of serological testing is not recommended by any of the societal groups for diagnosis or management of IBD at this time.

In the future, we can anticipate implementing multiomics and precision medicine in the management of IBD. Multiomics is the comprehensive analysis of biological data, which include genomic, epigenomic, transcriptomic, and proteomic data among others [18,19,20]. For example, the intestinal mucus structure is altered in IBD and can be studied using glycomics (the measurement of diverse glycan structures on cell walls) to gain insight into IBD pathogenesis. Emerging research in multiomics has the potential to identify the molecular pathways that drive IBD initiation, progression, and response to treatment. Furthermore, the profile of gut microbiota, or gut microbiome, is different in IBD [19]. While the biological significance of this is under investigation, gut microbiome analysis can contribute to the application of precision medicine and better management of patients in the future.

## 3. Laboratory Tests to Assess Disease Activity

Treatment endpoints for inflammatory bowel disease include improvement in symptoms, normalization of biochemical markers, and endoscopic mucosal healing. Among those, endoscopic mucosal healing has emerged as a superior endpoint because it correlates with long-term maintenance of remission and improved outcomes [21,22,23]. However, given the cost and inconvenience of frequent endoscopic procedures, it would be ideal to assess disease activity using biomarkers. Biomarkers are molecules that can be quantifiable in tissue, blood, stool, or urine and represent activity level, presence, or absence of disease processes [24,25]. The use of biomarkers to monitor disease activity, rather than endoscopic surveillance, is of particular importance for unique patient groups who are at high risk for endoscopic procedures, such as those with significant cardiopulmonary comorbidities, those who are pregnant, or those of advanced age. Abnormal biomarkers in patients with severe comorbidities and of advanced age can be due to these comorbidities rather than IBD, and the assessment of IBD disease activity requires an individual-based, detailed, and thorough approach. Similarly, inflammatory biomarkers can be elevated due to pregnancy rather than active IBD. Stool studies and blood tests are preferred in pregnant patients, as the benefit and risk of endoscopic or radiologic assessment of disease activity is not routinely performed and requires a careful risk–benefit analysis. Assessment of disease activity is also frequently performed using biomarkers in children, as endoscopic assessment is less frequently performed. The biomarkers that have been best studied in the assessment of IBD disease activity include C-reactive protein (CRP), fecal calprotectin (FCP), and fecal lactoferrin.

### 3.1. C-Reactive Protein

C-reactive protein (CRP) is an acute-phase protein produced by the liver in response to the pro-inflammatory cytokine IL-6. Elevations in CRP are not specific to intestinal inflammation and can be seen with other inflammatory conditions and infections. Age and sex have been independently associated with CRP levels [26]. Moreover, genetic variations in the expression of CRP may result in some patients with active disease to have lower degrees of CRP elevation [27]. Thus, an elevated CRP should heighten suspicion for active disease, especially when present in conjunction with symptoms of IBD flare, but is not sufficiently sensitive or specific to be used alone. Its utility is to be used as an adjunct to endoscopy, imaging, or other laboratory tests, with confidence increased when CRP is followed longitudinally and known to have good correlation with endoscopically confirmed disease activity [24,25]. In hospitalized patients with known active disease, CRP has a distinct benefit. CRP has a half-life of 19 h, so when elevated it can be used to trend clinical progression on a daily basis [28]. CRP can be easily obtained with daily bloodwork in most hospital settings and is relatively inexpensive and quick to demonstrate results. The most recent guidelines by the American Gastroenterology Association (AGA) recommends the use of CRP as a biomarker together with other biomarkers every 6–12 months in asymptomatic individuals and more frequently (for example every 1–4 months) and together with clinical assessment in symptomatic patients, although routine use of CRP for the assessment of postoperative recurrence of CD is not recommended [24,25].

### 3.2. Fecal Calprotectin

Fecal calprotectin (FCP) is a protein present within the cytoplasm of neutrophils. Active intestinal inflammation results in the migration of neutrophils to the intestinal wall and leakage of calprotectin into the intestinal lumen, which is measurable within the stool. Elevations in FCP levels correlate well with endoscopic disease activity but are most sensitive in patients with extensive colonic disease [29]. In UC, FCP is less sensitive in patients with proctitis compared to patients, who have left-sided colitis or pancolitis [30]. In CD, some studies have suggested inferior performance of FCP in detecting small bowel inflammation compared to colonic disease [25,31]. This is because small bowel inflammation tends to produce less robust elevations in FCP compared to colonic inflammation. It has been proposed that lowering the test cutoff of FCP for small bowel CD may improve its sensitivity, with one meta-analysis showing that sensitivity increased from 80% to 92% when the cutoff was reduced from 100 μg/g to 50 μg/g, but the tradeoff is reduced test specificity [32]. As with CRP, confidence in FCP can be increased for a particular patient by following their values longitudinally and historically, noting good correlation with endoscopically confirmed active disease and remission. While FCP is generally more specific for intestinal inflammation than CRP, it can become elevated in settings of gastrointestinal infection. Therefore, in patients with symptoms of IBD flare and elevated FCP, it is advised to obtain stool testing for *Clostridium difficile* infection and other enteric pathogens. The AGA guidelines on the use of biomarkers in CD and UC suggest that an FCP threshold of 150 μg/g in the appropriate clinical context may be sufficient to determine the presence of active disease. In CD patients with bowel resection, surveillance with a lower cutoff of 50 μg/g is recommended [24,25]. Overall, FCP is recommended as a cost-effective test that reflects intestinal inflammation.

### 3.3. Fecal Lactoferrin

Fecal lactoferrin is a protein present within neutrophil granules, and its presence in stool is proportional to the extent of neutrophil migration into the intestinal lumen. It has a performance similar to FCP, with good sensitivity for intestinal inflammation but without the ability to distinguish inflammation due to IBD from other etiologies. Its sensitivity is improved by extensive colonic disease inflammation and, as a result, may perform better in UC than CD [33].

Additional serum and fecal biomarkers have been proposed. The erythrocyte sedimentation rate (ESR) is frequently obtained as an indirect measure for inflammation but has shown poorer performance than CRP [34]. Other acute-phase reactants may be elevated in active disease, resulting in observations of thrombocytosis, anemia, and hypoalbuminemia, but these are not sensitive nor are they specific enough for surveillance in asymptomatic individuals. The Endoscopic Healing Index (Prometheus Monitr^®^, Prometheus Laboratories, San Diego, California, USA) is a proprietary test that detects 13 serum proteins to produce a quantitative score ranging from 0 to 100 units, with a score < 20 units validated to rule out active inflammation in CD [35]. Its use has been extended to UC but has been slow to gain traction due to its additional cost and no improved accuracy over FCP [36]. More recently, serum free thiols, which are reflective of oxidative stress, have been reported to have improved sensitivity over CRP, though not FCP [37]. Fecal markers S100A12, polymorphonuclear elastase, alpha-1-antitrypsin, and M2-pyruvate kinase have also been reported but are not widely used in practice [38]. Fecal occult blood testing is not routinely used due to poor sensitivity compared with FCP and fecal lactoferrin, as intestinal bleeding is a late symptom of severe IBD [39]. Newer biomarkers, for example, leucine-rich alpha-2 glycoprotein (LRG), whose blood level was shown to correlate with disease activity in UC or microRNA molecules that are differentially expressed in blood or tissue, are under investigation for use in the assessment of IBD disease activity [40]. Nonetheless, the sensitivity and specificity of these newer biomarkers remain to be established.

Biochemical markers should be obtained at diagnosis and routinely thereafter for the surveillance of disease activity [41]. The ideal monitoring interval and combination of biomarkers to use are yet to be determined. The European Crohn’s and Colitis Organization (ECCO) recommends obtaining FCP at diagnosis and every 3–6 months after initiation of treatment [41]. The AGA recommends monitoring biochemical markers every 6–12 months in patients who have achieved remission, with preference for stool-based tests due to better correlation with mucosal healing [24,25]. Aside from the development of new symptoms, the assessment of disease activity may also be prompted by evidence of new or worsening anemia, as bleeding lesions in the GI tract cause iron deficiency, and active inflammation is associated with anemia of chronic disease.

## 4. Therapeutic Drug Monitoring

Therapeutic drug monitoring (TDM) is a traditional concept in clinical medicine with routine measurements of in vivo drug concentrations for medications with a narrow therapeutic window, such as digoxin or gentamycin, because these medications are ineffective below and toxic above a certain blood concentration. TDM is also widely used in transplant patients, in whom immune suppression is necessary to achieve a therapeutic drug concentration to prevent rejection and avoid toxic blood levels, especially for critical-dose drugs with a narrow therapeutic window, such as tacrolimus, sirolimus, and mycophenolate mofetil. In regard to IBD, in vivo concentrations of azathioprine metabolites were found to correlate with response and high concentrations with toxicity, as we will discuss below. For biologics, whereas high blood concentrations were feared to be associated with possible toxicity, in practice higher drug concentrations have correlated positively with clinical response. This correlation of drug concentration with clinical efficacy has been confirmed by several randomized controlled trials, as we will discuss below. The measurement of serum drug concentrations has been investigated as a potential clinical tool to optimize therapy. Therapeutic drug monitoring (TDM) is usually performed for patients using immune modulators or biologics, where the latter include monoclonal anti-TNF, anti-integrin, anti-IL12 (p40), and anti-IL23 (p19) antibodies. Small molecules, such as sphingosine 1-phosphate (S1P) receptor modulators or Janus kinase (JAK) inhibitors, are not routinely monitored with TDM due to the limited availability of evidence.

### 4.1. Therapeutic Drug Monitoring for Immune Modulators

Immune modulators include thiopurines (azathioprine and 6-mercaptopurine (6-MP)) and methotrexate (MTX). Although effective as monotherapy, their narrow therapeutic window is associated with complications during long-term use [42]. However, immune modulators are attractive medications when used in combination with biologics for two reasons: (1) they reduce antibody formation against biologic agents, and (2) there is benefit from the additive therapeutic effect of an immune modulator when used in combination with a biologic [43]. Whereas therapeutic drug monitoring is not routinely indicated for MTX use, laboratory monitoring is performed for thiopurine therapy both prior to and during treatment (Table 1).

Expression levels of two enzymes, thiopurine methyltransferase (TPMT) and nudix hydrolase-15 (NUDT-15), have a strong impact on the metabolism of thiopurines and accumulation of toxic metabolites [46]. Low activity of these enzymes in the blood predisposes patients to myelosuppression and severe leukopenia. Therefore, testing for TPMT and NUDT-15, either by genotyping or phenotyping, can identify poor metabolizers who may benefit from either alternative therapy or lower medication doses. However, the etiology of myelosuppression and leukopenia in these patients is not entirely clear, and most cases of leukopenia occur in patients with normal enzyme activity and during the first several months after starting therapy [47]. Therefore, routine monitoring of a complete blood count (CBC) with differential constitutes a more important routine than quantitation of TPMT in patients with IBD. As TPMT enzyme activity does not reliably predict a leukopenia response, its quantitation is not recommended in guidelines of the AGA or ECCO; however, it is recommended in the guidelines of the United States Food and Drug Administration [44]. Based on the frequent presence of disease-causing variants in Asians, the expression of NUDT-15 variants are only screened in Asians before starting thiopurine therapy [45].

Thiopurine metabolite monitoring is not routinely performed but may be prompted by either poor therapeutic response or evidence of drug toxicity. The most common drug toxicities are myelosuppression and hepatotoxicity, both of which should be monitored by routine CBC and liver enzyme testing. Thiopurines are metabolized into two important molecules: 6-thioguanine nucleotide (6-TGN) and 6-methyl mercaptopurine (6-MMP). A 6-TGN level between 230 and 450 pmol/8 × 10^8^ erythrocytes is associated with clinical response, whereas lower doses can be associated with poor clinical outcome and require dose escalation [48]. Although higher levels of 6-TGN can cause myelosuppression, patients with high levels of 6-TGN without evidence of myelosuppression should not receive dose reductions in setting of a beneficial clinical response to therapy. In other words, we do not de-escalate therapy in asymptomatic patients solely due to the presence of elevated 6-TGN levels. Hepatotoxicity can occur when 6-MMP levels are elevated above 5700 pmol/8 × 10^8^ erythrocytes. We recommend a thorough etiological workup for elevated transaminase levels, and if hepatotoxicity is believed to be due toxic accumulation of thiopurine metabolites, the medication dose can be reduced or changed to another agent. An elevated 6-MMP/6-TGN ratio exceeding 20 is associated with poor clinical response and increased toxicity; therefore, these patients should be offered alternative therapy [49,50,51]. As cohorts have found an increase in 6-MMP and a decrease in 6-TGN level during pregnancy, these metabolites are recommended to be measured prior to conception, as this can help with the clinical assessment in case the patient has a flare during pregnancy [52].

### 4.2. Therapeutic Drug Monitoring for Biologics

Biologics, which include monoclonal anti-TNF, anti-integrin, anti-IL12/IL23 (p40), and anti-IL23 (p19) antibodies, have revolutionized IBD treatment in the last 10–20 years by effectively inducing clinical and endoscopic remission and achieving better symptom control. Still, treatment failures are common with biologics. TDM for biologics involves the measurement of drug trough levels and anti-drug antibodies (ADA) and can guide drug management when treatment failures occur.

Primary treatment failure occurs if the patient does not respond to a specific biologic therapy after beginning it. This occurs in one-third of patients initiated on a biologic, with about 20–30% of these failures attributed to inadequate dosing or drug levels [53,54]. Numerous studies have documented favorable outcomes with higher induction, postinduction, and maintenance anti-TNF drug levels [55,56], including post hoc analysis of the CLASSIC I/II trials, which found a positive association between adalimumab trough concentrations and clinical remission at week 4 [57]. The association of higher trough levels and improved outcomes remains apparent in more severe manifestations of IBD, such as fistulizing CD and acute severe UC [58,59]. Accordingly, low drug concentrations have been associated with primary non-response and immunogenicity, as demonstrated in the PANTS study, a prospective observational study involving CD patients treated with infliximab and adalimumab [60]. Measurement of drug trough levels are traditionally obtained immediately before the next dose of medication is administered. However, the POETIC study suggests that the trough level of adalimumab is relatively stable throughout the injection cycle and that timing is of little importance [61]. Recently, the development of the proprietary Prometheus Predictr^®^ test (Prometheus Laboratories, San Diego, California, USA) allows for the estimation of infliximab and adalimumab trough levels regardless of laboratory timing [62]. Oftentimes adjustments in dosing and frequency of biologic administration can increase drug trough levels if desired.

In contrast, secondary treatment failure occurs if the patient loses response to a biologic after benefiting from treatment for a while. Approximately 40% of patients initiated on an anti-TNF will lose response within the first year, which is often prompted by the formation of ADA [63]. ADA resulted from host development of immunogenicity against a biologic protein molecule. ADA may prevent binding of the drug to its target molecule or may promote rapid clearance of the drug through the formation of immune complexes. Ultimately, ADA reduces the effectiveness of the biologic and results in clinical loss of response [64]. While development of immunogenicity is typically associated with secondary treatment failure, rapid onset can be observed after the initiation of adalimumab therapy. The POETIC study demonstrated that over half of the cases of adalimumab immunogenicity occurred within the first 2 weeks of therapy [61]. When a treatment failure occurs due immunogenicity, a change in drug class of therapy is required.

It is worth mentioning that individual disease or inflammatory states can change the sensitivity or responsiveness of the patient to a biologic. Biologics are believed to target inflammatory pathways, yet clinical practice frequently does not quantitate the activity of these pathways. If we take inflammation as a whole as a target, individual patients may present in different inflammatory or disease states, or their inflammatory state can change as the disease evolves. For example, laboratory parameters of patients, such as low albumin, can affect trough level and thus the efficacy of the medication [65,66].

### 4.3. Therapeutic Drug Monitoring for Small-Molecule Drugs

Small-molecule drugs used in IBD treatment are sphingosine-1 phosphate receptor (S1P receptor) modulators and Janus kinase (JAK) inhibitors. The literature for the TDM of small-molecule drugs is limited. There are no clinically available drug assays to assess drug levels of the JAK inhibitors. Although there is an assay to measure active metabolites of ozanimod, an S1P receptor modulator, the safety of dose escalation has not been determined, and there is greater potential for bradycardia with higher doses [67].

### 4.4. Evidence for Therapeutic Drug Monitoring

There is evidence supporting the use of TDM in IBD management to optimize therapy, yet how extensively TDM should be used in routine practice is not well defined. There are two strategies commonly employed. First, TDM is performed in a proactive manner, where regardless of clinical response to the medication, the drug trough level is measured and adjusted to reach a target concentration. Second, TDM is performed in a reactive manner where the drug concentration to a medication is measured with ADA after a patient fails to respond to a medication or the response is less than expected. Several prospective and retrospective studies have been published on the topic of TDM in IBD, and limited evidence indicates that proactive TDM offers reproducible benefit to patient management [55,68,69]. Landmark studies and outcomes are listed in Table 2 and are briefly summarized below.

The TAXIT study published in 2015 randomized CD and UC patients to receive infliximab maintenance dosing directed by either clinical response (symptoms and CRP) or TDM (target drug trough 3–7 μg/mL) [70]. The primary endpoint of clinical and biochemical remission at one year was similar between groups (66% in the clinical response group vs. 69% in the TDM group, *p* = 0.686), although the TDM group had fewer flares during the course of treatment. Subsequently in 2018, the TAILORIX randomized controlled trial failed to show benefits of proactive drug monitoring using biochemical markers of inflammation (CRP, FCP) and drug trough levels over dose escalation based on clinical symptoms alone for patients with CD receiving infliximab maintenance therapy [71]. Patients randomized to drug monitoring either received dose escalation of infliximab by intervals of 2.5 mg/kg (TDM group 1) or directly to 10 mg/kg (TDM group 2). The primary endpoint of corticosteroid-free remission weeks 22 through 54 was similar between groups (33% in TDM group 1 vs. 27% in TDM group 2 vs. 40% in control group, *p* = 0.50).

The SERENE-CD randomized controlled trial published in 2022 showed that a higher induction regimen for adalimumab was not superior to a standard induction regimen [76]. The primary endpoints of clinical remission at week 4 (44% for both, *p* = 0.939) and endoscopic remission at week 12 (43% in the high-induction group vs. 39% in the control group, *p* = 0.462) were similar between treatment groups. Of note, after week 12, patients were re-randomized to receive maintenance adalimumab dosing using either TDM or clinical response alone (symptoms and CRP), and both groups showed similar efficacy and safety. While these landmark studies failed to show a clear benefit of using TDM, the same studies and numerous others have documented favorable outcomes in the context of higher induction, postinduction, and maintenance anti-TNF drug levels [55], including post hoc analysis of the CLASSIC I/II trials, which found a positive association between adalimumab trough concentrations and clinical remission at week 4 [57]. The association of higher trough levels and improved outcomes remains apparent in more severe manifestations of IBD, such as fistulizing CD and acute severe UC [58,59].

Accordingly, low drug concentrations have been associated with primary non-response and immunogenicity, as demonstrated in the PANTS trial, a prospective observational study involving CD patients treated with infliximab or adalimumab [60]. The POETIC prospective observational study from 2018 elucidated the high frequency of ADA with biologic therapy. Among CD patients treated with adalimumab, ADA developed in one-third of patients, with the majority of these cases occurring within the first 14 weeks of therapy [61]. The authors suggest the need for tighter TDM during the first months of therapy when the development of ADA is most likely, followed by less frequent monitoring over time as this risk decreases.

**Figure 2:**
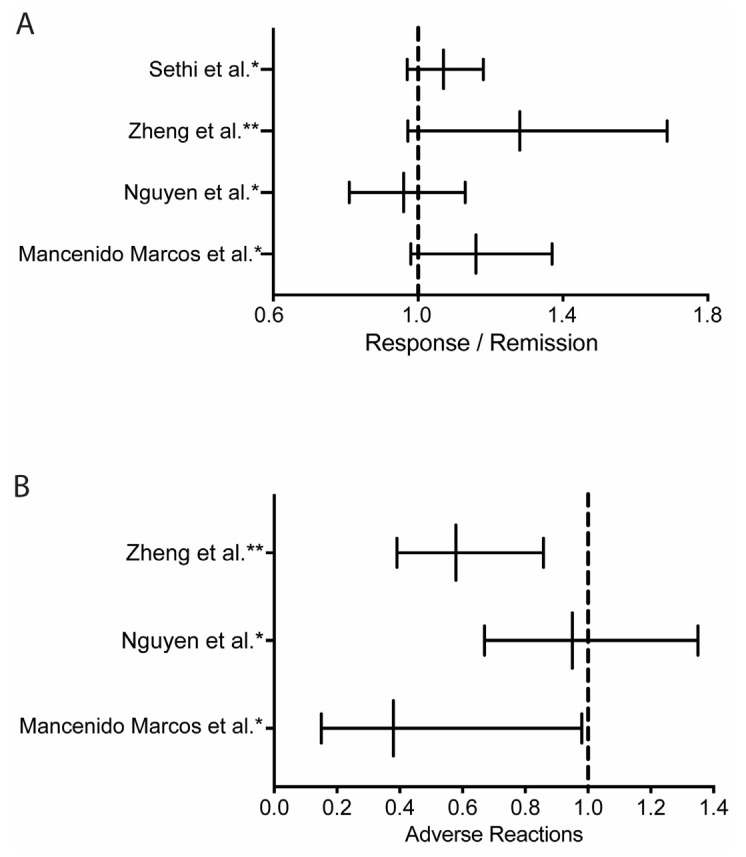
Forest plot display of metanalysis results on the impact of TDM on clinical response/remission (**A**) and adverse reactions (**B**). Data are adapted from data sets of recent metanalyses [68,69,78,79] and displayed as either relative risk (RR) * or odds ratio (OR) ** with confidence intervals.

**Figure 3:**
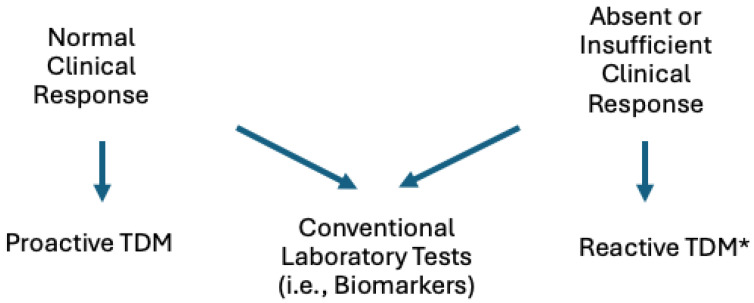
Selective tools (each arrow) to optimize biologic therapy in different clinical scenarios. * Reactive TDM is a generally accepted clinical approach.

TDM has also been addressed by several recent meta-analyses that did not find an overall improvement in rates of clinical remission with use of proactive TDM for anti-TNF therapy (Table 3 and Figure 2) [68,69,78,79]. However, secondary and subgroup analyses did find differences between groups—for example, lower rates of adverse events and complications with proactive TDM in some studies (Figure 2). Zheng et al. showed that proactive TDM resulted in lower rates of acute infusion reactions and delayed hypersensitivity reactions (OR: 0.579; 95% CI: 0.391–0.858; I^2^: 67.2%) [79]. A similarly decreased rate of adverse reactions was demonstrated in a recent metanalysis by Marcos et al. [78] (Figure 2). In addition, Sethi et al. showed that proactive TDM was associated with lower rates of treatment failure (RR: 0.46; 95% CI: 0.21–0.98; *p* < 0.05) and the need for hospitalization (RR: 0.33; 95% CI: 0.21–0.54; *p* < 0.01) [68]. Although TDM can be regarded to be intuitively built on treat-to-target strategies (Selecting Therapeutic Targets in Inflammatory Bowel Disease (STRIDE) Initiative) in IBD, which include clinical improvement, mucosal healing, or normalization of CRP, TDM is not included in these parameters [25,80,81,82]. For now, it is the authors’ opinion that TDM is a single tool among several that clinicians can use to assess clinical response (Figure 3). Although future studies can attest to a benefit of using proactive TDM in different clinical scenarios—such as the potential to improve safety and cost-effectiveness [68], and especially in situations where dose de-escalation may be performed reducing drug exposure and cost [83]—for now we do not recommend the routine use of proactive TDM in daily practice until solid and confirmatory data from more standardized clinical trials, observational studies, or metanalyses emerge.

Less is known about the use of TDM for biologics outside of the anti-TNF drug class. Vedolizumab, a monoclonal antibody against α4β7, was shown to induce clinical response at higher rates among patients who achieved the highest drug trough levels in GEMINI I and GEMINI II trials [84,85]. However, in the TUMMY prospective, observational study published in 2023, higher vedolizumab trough levels were associated with biochemical remission but not with clinical remission during maintenance treatment [86]. Regarding anti-interleukin class drugs, a recent meta-analysis showed that higher drug trough levels of ustekinumab correlated with higher rates of clinical and endoscopic remission during maintenance treatment [87]. Similar findings are reported for risankizumab [67]. Despite awareness that optimal drug trough levels may improve clinical outcomes, the role of TDM is not yet established for anti-integrin and anti-interleukin classes of drugs. Unlike with anti-TNF drugs, rates of ADA against vedolizumab, ustekinumab, and risankizumab are relatively low at less than 5% [67,88,89].

### 4.5. Societal Guidelines on TDM

Societal guidelines generally promote use of reactive TDM over proactive TDM. In 2017, the AGA promoted reactive TDM as a tool for IBD treatment decisions but made no suggestion regarding proactive TDM [90]. A study from the same year found that reactive TDM significantly reduced treatment costs [91]. In 2021, the American College of Gastroenterology (ACG) published a consensus statement through a comprehensive literature review and a modified Delphi method, which agreed on the use of reactive TDM for all biologics for both primary and secondary nonresponse and proactive TDM for anti-TNF therapy during induction, at least once during maintenance, and after a long drug holiday [92]. ECCO recommends reactive drug monitoring, with best evidence of proactive drug monitoring among anti-TNF class biologics [88].

### 4.6. Anti-TNF-Induced Lupus

The use of biologics are associated with specific side effects that may be life threatening, where the management requires laboratory tests. A special form of anti-drug antibodies that can arise in patients treated with anti-TNF therapy is anti-TNF-induced lupus (ATIL). This is a hypersensitivity reaction that can manifest with symptoms of idiopathic systemic lupus erythematosus like arthritis, rash, pleuritis, and pericarditis [93]. Diagnosis is confirmed by the classical laboratory pattern of positive antinuclear antibodies (ANA), positive anti-double-stranded-DNA (anti-dsDNA), and negative anti-histone antibodies in the right clinical setting. ATIL differs from classical drug-induced lupus due to its lower frequency of anti-histone antibodies, 57% vs. >95% reported in one case series [94]. The sensitivity of ANA (>99%) is greater than anti-dsDNA (90%); therefore, the diagnosis of ATIL may still be made in the presence of a negative anti-dsDNA if clinical suspicion remains high. Stopping the medication that can cause drug-induced lupus is essential, although steroids or immune suppressives can be used to treat various complications including life-threatening ones. Once ATIL is diagnosed, anti-TNF therapy should be discontinued indefinitely.

## 5. Laboratory Tests to Diagnose and Prevent Infections

### 5.1. Diagnosis of Concomitant Gastrointestinal Infections

IBD and immune suppressive therapy are independent risk factors for infectious diseases, which include gastrointestinal infections. As part of the diagnostic evaluation of precipitating factors of an acute IBD flare in the presence of new onset symptoms, gastrointestinal infections should be excluded. Stool specimens should be collected to test for routine enteric pathogens including *Clostridium difficile* [41]. Cytomegalovirus (CMV) colitis should be considered in the immunosuppressed patient and may be detected in 30% of cases of steroid-refractory UC [95]. The diagnosis of CMV colitis requires tissue acquisition, as the detection of CMV in blood and stool samples is less sensitive [96]. Nevertheless, blood polymerase chain reaction (PCR) testing may detect viremia associated with disseminated CMV and if present may be used to noninvasively monitor response to therapy. Thus, a combination of both blood- and tissue-based tests may be used to assess for CMV infection.

### 5.2. Screening for Chronic Infections Prior to the Initiation of Immune Suppressive Therapy

Patients treated with immune suppressive therapy are prone to developing severe and life-threatening infections, where the prevention and treatment of certain infections belong to strategic targets (Figure 1). Opportunistic infections like histoplasmosis, Epstein–Barr Virus (EBV), and *Pneumocystis jirovecii* are complications of chronic immune suppressive therapy. Additionally, reactivation of chronic infections such as tuberculosis and HBV can occur after initiation of immune suppressive therapy, especially with anti-TNF class drugs. Clinical vigilance is required to diagnose these infections in a timely manner as routine screening is not recommended, with tuberculosis and HBV being notable exceptions.

The etiological agent of tuberculosis is *Mycobacterium tuberculosis*, which can cause severe and life-threatening infections in the lung and other organs. Although *Mycobacterium tuberculosis* causes a silent and dormant infection in the majority of cases, IBD patients on biologics have a 14-fold higher risk to develop primary severe mycobacterial infection or reactivation [97]. Therefore, patients should be screened for latent tuberculosis infection prior to starting and during immune suppressive therapy. This screening is performed preferably with in vitro interferon-γ release assay (IGRA) of cultured T lymphocytes that are activated by mycobacterial antigens (QuantiFERON-TB Gold test) [98]. Tuberculin skin test (TST) is another alternative but should not be used in patients with prior BCG vaccination due to false positive results. Treatment is usually indicated for a positive IGRA test result, for which we recommend consultation with an infectious disease specialist to direct antimycobacterial treatment in this immune-suppressed population. Occasionally a patient with severe IBD symptoms requires steroid treatment prior to obtaining IGRA testing, which carries an up to 11% risk of an indeterminate result and can also result in a false negative result on TST [99]. The interpretation of such results is difficult, so ideally patients should be screened for TB prior to onset of acute illness.

HBV can cause severe and fulminant hepatitis in immune-suppressed individuals. This can occur in the form of reactivation of HBV in a chronic carrier or a new infection. IBD patients on immune suppression are screened for HBV infection by testing hepatitis B virus surface antigen (HBsAg) in their blood and for immunity against HBV by quantitating the antibodies against HBsAg (anti-HBs). Immunity against HBV can occur after vaccination or natural infection. The laboratory difference between these two forms of immunity is the presence of antibodies against HBV core antigen (anti-HBc), which is important in clinical practice because immunity after natural infection can be associated with persistence of a low level of HBV replication in immune-privileged sites in the body, which can reactivate in immune-suppressed individuals (i.e., patients receiving rituximab) and cause severe hepatitis [100]. Therefore, we recommend including anti-HBc serology when screening for HBV infection in IBD patients and to follow up a positive anti-HBc serology with serum HBV DNA quantitative PCR. If present, HBV should be treated prior to the initiation of immune suppressive therapy, although activation is not common in IBD patients compared with rituximab recipients [101].

In pediatric patients treated with immune modulators, a new infection with Epstein–Barr virus (EBV) can cause life-threatening lymphoproliferative disorders. Therefore, pediatric patients are recommended to be screened for past EBV exposure with serology prior to the initiation of immune modulator therapy. Thiopurines are not recommended in EBV-naïve children.

## 6. Assessment of Nutritional Deficiencies

Deficiencies in iron, vitamin B12, folic acid (vitamin B9), and vitamin D are frequently observed in patients with IBD due to malabsorption and poor nutritional intake. Therefore, the assessment of nutrition and detailed management of vitamin and mineral deficiencies are strategic targets of IBD management (Figure 1). Patients should receive routine monitoring for nutritional deficiencies, and abnormalities should be corrected when found. Current AGA guidelines recommend that all patients with IBD should be screened for iron and vitamin D deficiency, and those with ileal resection or extensive ileal disease should also be screened for vitamin B12 deficiency [102]. ECCO guidelines additionally recommend screening for folic acid deficiency in patients with small bowel CD [41]. The frequency of monitoring is left to clinical judgment, with shorter frequency (i.e., 3–6 months) favored in settings of active disease, with small bowel involvement, or after small bowel resection [41]. Laboratory assessment of nutritional balance and deficiencies are very important in pediatric patients, as deficiency can affect growth.

### 6.1. Iron

The presence of microcytic anemia should prompt evaluation for iron deficiency. Even in the absence of anemia, iron stores should be routinely assessed in patients with IBD because iron deficiency can indicate ongoing subtle disease activity, and the replacement of iron contributes to the improvement of a patient’s overall well-being. The diagnosis of iron deficiency is dependent on the level of inflammation present since ferritin is an acute phase reactant. In absence of inflammation, ferritin levels < 30 μg/L are diagnostic, whereas the presence of inflammation may raise this threshold to <100 μg/L [41]. The measurement of soluble transferrin receptor can help differentiate between anemia of chronic disease and iron deficiency, where soluble transferrin receptor levels are low or normal in anemia of chronic disease and high in iron deficiency. Newer iron indices that may alternatively be used include reticulocyte haptoglobin content, zinc protoporphyrin, and the percentage of hypochromic red cells [103].

### 6.2. Vitamin B12

Vitamin B12 is a water-soluble vitamin that is selectively absorbed in the terminal ileum. The absorption of vitamin B12 is impaired in patients with terminal ileal disease or after ileal resection. Patients with IBD are also prone to developing small intestinal bacterial overgrowth, which predisposes them to vitamin B12 deficiency [104]. Vitamin B12 deficiency is common but may be underdiagnosed because serum blood measurements tend to overestimate stores. The measurement of methylmalonic acid levels is more sensitive [105]. Vitamin B12 deficiency can result in megaloblastic anemia and peripheral neuropathy, and severe deficiency is associated with subacute combined degeneration, which may result in permanent neurologic defects unless promptly diagnosed and treated.

### 6.3. Folic Acid

Folic acid (vitamin B9) is a water-soluble vitamin absorbed in the duodenum and jejunum [106]. Folic acid deficiency can be multifactorial due to inadequate dietary intake, malabsorption, or iatrogenic in the setting of sulfasalazine and methotrexate therapy [107]. Ileal CD has been shown to be a risk factor for folic acid deficiency [108]. Supplementation can prevent complications of folic acid deficiency such as the development of megaloblastic anemia and fetal neural tube defects during pregnancy.

### 6.4. Vitamin D

Vitamin D is essential to calcium homeostasis and contributes to the proper functioning of the immune system. Vitamin D is a fat-soluble vitamin, and its absorption can be reduced in patients with malabsorption from intestinal diseases such as IBD. One recent prospective study from Taiwan detected vitamin D deficiency in 42% of patients with IBD [109]. Another metanalysis has shown that risk factors for vitamin D deficiency include being of a non-Caucasian race (OR: 3.79, 95% CI: 2.68–5.34), having a diagnosis of Crohn’s disease (OR: 1.38, 95% CI: 1.21–1.56), having an increase in IBD disease activity (OR: 1.85, 95% CI: 1.61–2.13), having a history of IBD-related resection (OR: 1.61, 95% CI: 1.38–1.89), the use of steroids (OR: 161, 95% CI: 1.28–2.03) or biologics (OR: 1.78, 95% CI: 1.48–2.14), and being a member of the male sex (OR: 1.84, 95% CI: 1.47–2.31) [110]. Interestingly, vitamin D deficiency is frequently diagnosed prior to onset of IBD, which supports the theory that vitamin D has a role in the regulation of immunity and maintenance of the intestinal barrier function [111]. In mouse models, the administration of vitamin D3 in diet has been shown to improve colitis severity [112,113]. A Danish randomized controlled trial in which vitamin D3 (1200 IU daily) was administered to patients with CD showed a trend towards lower rates of clinical relapse compared to placebo, although it did not reach statistical significance [114]. Regardless, supplementation of vitamin D in deficient patients will certainly improve bone metabolism and reduce risk for developing osteoporosis [115].

Besides the screening recommendations above, patients should be screened for hypomagnesemia and hypocholesterolemia before being started on cyclosporin therapy to prevent severe neurotoxicity [116]. While historically the monitoring of serum proteins such as albumin were used to identify malnutrition, this is no longer recommended due to poor sensitivity, and these protein levels are influenced by active inflammatory states [102].

## 7. Conclusions

Laboratory tests are frequently utilized in the management of patients with IBD, ranging from diagnosis to therapeutic drug monitoring and beyond. In summary, laboratory tests for genetic, serologic, and metaomics are on the horizon but do not yet have a role for clinical use. Common laboratory tests for the assessment of disease activity include CRP, FCP, and fecal lactoferrin, with preference for stool-based testing due to its improved specificity. TDM for immune modulators can identify patients at risk of developing toxicity, while TDM for biologics is mostly limited to anti-TNF drugs in settings of treatment failure. Labs should be obtained to screen for tuberculosis and HBV prior to the initiation of immune suppressive therapy, and they should also be obtained routinely to screen for nutrient deficiencies in patients with IBD. Due to their widespread accessibility, good patient tolerance, and low cost, laboratory tests serve as useful adjuncts in the assessment of IBD.

## Figures and Tables

**Table 1:** Laboratory tests used for TDM of immune modulators.

Therapeutic Drug Monitoring for Thiopurine Immune Modulator Therapy		
Compound	Rationale	Recommendation
Thiopurine Methyltransferase (TPMT)	Low activity of this enzyme predisposes patients to myelosuppression and leukopenia	Quantitation is not recommended in guidelines of the AGA but is recommended in guidelines of the US Food and Drug Administration [44].
Nudix hydrolase 15 (NUDT-15)	Low activity of this enzyme is linked to myelosuppression, particularly in Asians	Screening for variants is indicated in Asian patients prior to initiating thiopurine therapy [45].
6-thioguanine nucleotide (6-TGN)	Higher levels of this metabolite may cause myelosuppression	Monitor 6-TGN levels during therapy. Maintain levels between 230 and 450 pmol/8 × 10^8^ erythrocytes for effective treatment. If levels are low, adjust the dose to improve response.
6-methyl mercaptopurine (6-MMP)	High levels can cause hepatotoxicity	6-MMP levels above 5700 pmol/8 × 10^8^ erythrocytes is associated with development of hepatotoxicity and may warrant dose reduction or change in therapy.

**Table 2:** Landmark trials and observational studies (*) in adult and pediatric (**) IBD populations addressing the outcome of TDM for biologics.

Therapeutic Drug Monitoring in IBD—Landmark Biologic Trials						
Trial Name	Year	Drug	Study Design	Dose Schedules	Endpoint(s)	Key Findings
TAXIT [70]	2015	Infliximab	RCT comparing proactive TDM vs. standard care for UC and CD	Initially modified for all patients to a target trough concentration (TC) of 3–7 μg/mL. Afterwards, patients were randomized to dosing based on TC vs. clinical features	Primary: Clinical and biochemical remission within one year	TDM-based dose adjustment was associated with fewer flares, but the primary endpoint was not different between the two groups and therefore not reached
POETIC * [61]	2018	Adalimumab	Prospective observation for anti-adalimumab antibodies (AAA) in CD patients	Eligible patients were receiving the standard induction 160 mg at week 0 followed by 80 mg at week 2 loading course	Primary: Observe AAA formation in relation to clinical response Secondary: Observe AAA levels in relation to drug levels and TDM	AAAs are found to arise earlier than previously appreciated, indicating the importance of tighter TDM during the first months of therapy Drug and AAA levels were similar both at trough and in-between injections, enabling them to simplify TDM
TAILORIX [71]	2018	Infliximab	Multicenter prospective study comparing TDM-based dosing vs. clinical response in CD patients	(1) Increases (2 max) in steps of 2.5 mg/kg based on clinical course and drug concentration (2) Increase from 5 to 10 mg/kg based on the same criteria (3) Increase to 10 mg/kg based on clinical symptoms alone	Primary: Corticosteroid-free clinical remission	Primary endpoint was not different between groups and therefore not reached
PAILOT ** [72]	2019	Adalimumab	Nonblinded RCT of children with CD who were assigned to proactive and reactive groups	Initially scheduled to receive 40 mg in children weighing ≥40 kg or 25 mg/m^2^ body surface area in children < 40 kg. Then, randomized to groups of proactive vs. reactive monitoring with adjustments to achieve TC of 5 μg/mL	Primary: sustained corticosteroid-free clinical remission at all visits (week 8 through week 72)	Proactive monitoring of trough concentrations and dose adjustment resulted in significantly higher rates of corticosteroid-free clinical remission than reactive monitoring
PANTS * [60]	2019	Infliximab Adalimumab	Prospective observational study on CD patients treated with infliximab or adalimumab	The choice of anti-TNF was at the discretion of the treating physician and prescribed according to the licensed dosing schedule	Primary: treatment failure (primary non-response at week 14, non-remission at week 54, and adverse events leading to drug withdrawal)	Only factor independently associated with primary non-response and immunogenicity was low drug concentration at week 14. Continuing standard dosing regimens after primary non-response was rarely helpful
PRECISION [73]	2019	Infliximab	RCT comparing infliximab dosing guided by a Bayesian pharmacokinetic model, targeting the infliximab trough level of >3 μg/mL, to conventional treatment	(1) Control group continued maintenance treatment without dose adjustment (2) Received dosing based on a Bayesian PK model with a target trough level of 3 mg/mL Doses could vary between 1 and 10 mg/kg	Primary: proportion of patients in sustained clinical remission after 1 year	Model-based dosing was superior to conventional dosing in maintaining remission
NOR-DRUM [74,75]	2020	Infliximab	RCT comparing TDM vs. standard care for patients with varying autoimmune diseases including UC and CD	(1) Administration according to standard clinical care, without knowledge of drug levels or ADA status (2) After week 14, the dose or interval was adjusted to reach a target TC of 3–8 μg/mL	Primary: absence of disease worsening during the 52-week study period Secondary: efficacy, quality of life, drug survival, safety	Primary outcome of sustained disease control without disease worsening favored the TDM group
SERENE-CD [76]	2022	Adalimumab	RCT initially assessing higher vs. standard induction dose with further randomization after week 12 to dose optimization using the CDAI and CRP or TDM	(1) 160 mg at weeks 0–3 (2) 160 mg weeks 0–1, 80 mg weeks 2–4, 40 mg onward Rerandomization at week 12 to dose optimization using the CDAI and CRP or TDM	Co-primary: clinical remission at week 4 and endoscopic response at week 12	The clinical and endoscopic response was not superior in the TDM group, with a tendency of the TDM group showing poorer results
SERENE-UC [77]	2022	Adalimumab	Similar design as above with rerandomization at week 8	(1) 160 mg at weeks 0, 1, 2, and 3, 40 mg weeks 4 (2) 160 mg at week 0 and 80 mg at week 2, 40 mg weeks 4 Rerandomized at week 8 to 40 mg weekly, 40 mg every other week, or TDM	Co-primary: clinical remission (full Mayo score ≤ 2 with no sub-score > 1) at week 8 and week 52	Interpretation of the TDM results was limited but demonstrated clinical remission somewhere between high and standard dose

**Table 3:** Recent meta-analyses addressing the outcome of TDM for biologics.

Therapeutic Drug Monitoring in IBD—Meta-Analyses for Biologics						
Publication	Year	Included Studies	Inclusion Criteria	Intervention Studied	Endpoint(s)	Key Findings
Mancenido Marcos et al. [78]	2023	RCT (6), Systematic review (1), Cohort (2)	UC and CD patients receiving anti-TNF maintenance therapy	Proactive drug monitoring	Primary: Maintenance of clinical remission at 12 months Secondary: Safety and cost-effectiveness	Proactive TDM is not superior to conventional management (RR: 1.16, 95% CI: 0.98–1.37, I^2^: 55%). Proactive TDM was associated with improved safety (acute infusion reactions, drug discontinuation) and cost.
Nguyen et al. [69]	2022	RCT (9)	UC and CD patients receiving anti-TNF therapy	Proactive drug monitoring	Primary: Failure to maintain clinical remission Secondary: failure to maintain biochemical or endoscopic remission, risk of dose escalation, risk of discontinuation, risk of ADA, risk of achieving trough targets, and serious adverse events	Proactive TDM is not superior to conventional management with regard to primary outcome (RR: 0.96, 95% CI: 0.81–1.13, I^2^: 55%). Dose escalation was more likely to occur with proactive TDM, without additional benefit.
Sethi et al. [68]	2022	RCT (9), Cohort (15)	UC and CD patients receiving anti-TNF therapy	Proactive drug monitoring	Primary: Clinical remission and response Secondary: Treatment failure, need for surgery, endoscopic remission, need for hospitalization, and steroid-free remission	Proactive TDM is not superior to standard of care for achieving clinical remission or response (RR:1.07, 95% CI: 0.97–1.18, I^2^: 34%). Proactive TDM was associated with lower treatment failure over standard of care. Subgroup analyses favored proactive TDM over reactive TDM for preventing treatment failure and need for hospitalization.
Zheng et al. [79]	2024	RCT (4), Cohort (9)	UC and CD patients receiving anti-TNF therapy	Proactive drug monitoring	Co-primary: efficacy (clinical remission, need for surgery, treatment discontinuation, endoscopic remission, clinical relapse, ADA) and safety (total adverse events, acute infusion reactions, delayed hypersensitivity)	Proactive drug monitoring does not have superior efficacy over conventional management (OR: 1.281, 95% CI: 0.972–1.688, I^2^: 65.9%), but does have improved safety (OR: 0.579, 95% CI: 0.391–0.858, I^2^: 67.2%). Subgroup analyses did note greater clinical remission with proactive TDM in patients treated with adalimumab and when only RCTs were included. There were also lower rates of surgery with proactive vs. reactive TDM but not proactive vs. empiric TDM.

## Data Availability

No new data is generated for this review article. Please refer to the corresponding author of the citations for inquiries related to original data or archived datasets.
